# Resilience and academic performance in mathematics and science among middle school students: The mediating role of computational thinking

**DOI:** 10.1371/journal.pone.0360966

**Published:** 2026-10-27

**Authors:** Rumeysa Beyazhancer, Nurcan Kahraman, Barış Demir

**Affiliations:** 1 Bursa Uludag University, Faculty of Education, Department of Mathematics and Science Education, Bursa, Türkiye; 2 Kocaeli University, Faculty of Education, Department of Mathematics and Science Education, Kocaeli, Türkiye; PLOS: Public Library of Science, UNITED KINGDOM OF GREAT BRITAIN AND NORTHERN IRELAND

## Abstract

Psychological resilience has increasingly been recognized as a key factor associated with students’ academic performance, especially in demanding disciplines such as mathematics and science. However, limited research has examined the potential cognitive pathways associated with psychological resilience and academic achievement, particularly in subjects such as mathematics and science that require higher-order thinking skills. This cross-sectional and correlational study examined the indirect associations between psychological resilience, computational thinking (CT), and academic achievement in mathematics and science among middle school students. Data were collected from 401 middle school students in Türkiye. Psychological resilience was measured using the Brief Resilience Scale, CT was assessed via a 25-item multiple-choice test, and achievement scores in mathematics and science were obtained from official school records. Descriptive and correlational analyses were conducted using SPSS 28, while mediation analyses were performed in Mplus 8.1. Rasch analysis was applied to evaluate the psychometric properties of the CT instrument. Correlation results revealed significant positive associations among all main variables. Mediation analyses showed statistically significant indirect associations of psychological resilience with mathematics achievement through CT (β = .070, 95% CI [.036,.110]) and with science achievement through CT (β = .064, 95% CI [.026,.102]). After accounting for CT, the direct association between psychological resilience and mathematics achievement became nonsignificant, whereas the direct association with science achievement remained significant. The path model demonstrated an excellent fit. Overall, the findings suggest that CT may be statistically involved in the association between psychological resilience and academic achievement in mathematics and science; however, the cross-sectional design does not permit conclusions regarding temporal or causal mediation.

## Introduction

Science and mathematics are critical components of education systems worldwide, recognized not only for their role in developing essential cognitive skills but also for their contribution to a nation’s socioeconomic development and innovation capacity [1]. Despite widespread recognition of their importance, significant variation remains in student achievement across these disciplines [2]. Researchers increasingly emphasize the role of psychological factors in explaining these variations, highlighting psychological resilience as a particularly influential construct [3]. Psychological resilience can be defined as students’ ability to adapt and recover from challenges, such as academic failure, pressure, and uncertainty [4,5]. As educational systems increasingly recognize the importance of supporting learners not only cognitively but also emotionally and socially, resilience has gained prominence as a key predictor of academic engagement and persistence [3]. Students exhibiting greater resilience tend to navigate difficulties effectively, maintain motivation, and demonstrate sustained effort toward their academic goals [6].

This growing focus on resilience has significant implications for science and mathematics education, where students frequently face complex content, abstract reasoning, and heightened performance expectations. These disciplines often evoke anxiety, self-doubt, and fear of failure, making emotional coping and perseverance essential for success [7–10]. Studies show that resilient learners in science and mathematics settings are more open to exploration, more persistent in problem-solving, and more willing to learn from mistakes [11]. While the relevant literature has thoroughly explored various direct relationships between psychological resilience and academic achievement [9,10], according to the authors’ knowledge, the cognitive pathways underlying this relationship remain underexplored, particularly in science and mathematics. One construct that may help to explain this association is computational thinking (CT), which is a kind of problem-solving process that includes elements such as algorithmic thinking, logical reasoning, and abstraction [12,13]. Yet its potential role in linking psychological resilience to academic achievement has not been directly examined. Therefore, this study aims to investigate whether CT mediates the association between psychological resilience and student achievement in science and mathematics.

### Literature review and research questions

#### Psychological resilience.

The ability to cope successfully with significant change, difficulty, and distress is defined as psychological resilience [14]. This concept entered the academic fields of psychology and psychiatry in the 1970s. In the field of psychology, psychological resilience is commonly conceptualized through three core phenomena: (i) achieving positive outcomes despite exposure to significant risk, (ii) maintaining functioning under prolonged threat, and (iii) recovering from trauma [15,16]. These dimensions reflect an individual’s capacity to mobilize protective factors and adaptive processes when confronted with adversity. Accordingly, psychological resilience is commonly conceptualized both as a dynamic process and as a personal characteristic that supports individuals’ adaptation in the face of difficulties. Psychological resilience is the ability to preserve or restore psychological well-being when confronted with adversity, including emotional, cognitive, or environmental stressors [17–19]. Students’ emotional responses to academic challenges are influenced by the classroom environment. Recent investigations have demonstrated that the integration of humor into mathematics instruction can mitigate mathematics anxiety and subsequently foster greater student engagement in learning endeavors [20]. These supportive learning environments may, in turn, indirectly bolster students’ ability to manage challenges.

### Psychological Resilience and Academic Achievement

The relationship between psychological resilience and academic achievement has attracted increasing scholarly attention since resilience research entered the relevant literature. The Project Competence Longitudinal Study, an early longitudinal study led by Ann S. Masten in the 1970s and 1980s, explored how children demonstrate positive development despite adversity and identified key personal and environmental protective factors, such as cognitive competence and supportive parenting, that promote academic success and social adjustment. In other words, resilient students were those who were more likely to be academically successful despite facing risks [15]. Building on the foundational work of the Project Competence Longitudinal Study, numerous studies have explored the relationship between psychological resilience and academic achievement across various populations and educational contexts and affirmed the protective role of resilience in promoting academic success [21–25]. However, the relationship between psychological resilience and academic achievement is not consistently supported by the literature. Findings are mixed; while several studies suggest that psychological resilience has a significant impact on students’ academic performance in both the short and long term, as discussed above, other studies report no meaningful association between the two constructs [26–32]. For instance, Garcia-Martinez et al. found that resilience alone did not significantly predict academic performance among university students. However, their structural equation modeling revealed that self-concept acted as a mediating variable, linking resilience to academic success [33]. These findings emphasize the need to include additional psychological or cognitive factors to better understand the relationship between psychological resilience and academic achievement. This body of evidence suggests that resilience interacts with other constructs that may either mediate or moderate its influence on learning outcomes [34].

Psychological resilience may help students to sustain engagement with challenging mathematics and science tasks rather than disengaging after setbacks. Resilient students are more likely to persist through repeated failure, seek alternative strategies when an initial approach does not work, and view difficulty as a normal part of the learning process rather than a signal to withdraw. Research suggest that this determination is related to a more thorough investigation of different solution approaches and an increased openness to learning from errors [10]. In mathematics and science, where problem-solving is often iterative and requires tolerating initial failure, these tendencies not only parallel the practices at the core of CT, such as decomposition, algorithmic reasoning, and iterative debugging [35,36] but may also support students’ sustained engagement in them. However, this theoretical relationship remains largely unexamined in the existing literature. While investigations have demonstrated a positive association between CT skills and academic success, alongside advanced reasoning abilities in Science, Technology, Engineering and Mathematics (STEM) fields, to the best of the authors’ knowledge, no existing studies have examined CT as a mediating variable linking psychological resilience to performance in mathematics or science. CT is increasingly understood not simply as a technical skill but as a psychological—mainly cognitive—construct [37] that develops in close interaction with psychological factors; for instance, intrinsic motivation has been shown to be significantly related to students’ CT [38]. Therefore, this study examines whether CT mediates the relationship between psychological resilience and academic success, particularly in mathematics and science education.

### Computational thinking

CT is one of the most important 21st-century skills, recognized for its relevance across disciplines and its role in preparing learners for the demands of the digital age [13,39,40]. It was first introduced into education by the mathematician Papert [41]. The term CT education rooted in Papert’s work [42], has been used to emphasize the role of computers in promoting learning. Wing, who initially viewed CT as incompatible with education, later recognized it as a fundamental skill for the digital age and emphasized its integration into educational contexts [13]. She defined CT as “the cognitive processes involved in defining problems and developing solutions in such a way that they can be executed effectively by an information-processing agent” [43].

With the increasing interest in CT, initial research primarily focused on computer science education [12]. Subsequently, it was shown that CT could be meaningfully integrated into various educational disciplines, particularly mathematics and science education [44], leading to a growing body of research in these fields [45]. In line with this expansion, many mathematics and science educators have become increasingly engaged with CT, especially after its inclusion in the U.S. Next Generation Science Standards (NGSS) [46], where mathematical and CT is explicitly defined as a core practice of science and engineering [47]. Indeed, Wijaya et al. highlighted the importance of such cognitive processes within STEM education and pointed to the decisive impact of systematic thinking on academic outcomes [48].

Several CT assessment instruments have been developed, many of which incorporate the CT concepts, practices, and perspectives outlined in Brennan and Resnick’s framework [35]. For instance, researchers have designed instruments such as the Test for Measuring Basic Programming Abilities [49], the Commutative Assessment [50], and the Computational Thinking Test (CTt) [51]. These tests, as reviewed and discussed in Cutumisu et al. [52], represent notable efforts to operationalize CT and assess it across diverse educational contexts. The CT test designed by Gonzalez et al. has recently attracted considerable attention for its practicality and psychometric soundness [51]. In terms of the CT framework proposed by Brennan and Resnick [35], the CT test primarily focuses on computational concepts, while computational practices are represented to a limited extent and computational perspectives are not directly assessed. However, at the same time computational concepts such as sequencing, loops, and conditional structures represent the core cognitive mechanisms underlying CT. Previous studies indicate that algorithmic reasoning, logical sequencing, and control structures constitute fundamental components of CT development [12,53]. Although CT encompasses broader dimensions, including computational practices and perspectives [35], the CTt was selected because its emphasis on core computational concepts is well aligned with the objectives of the present study. The findings of the present study should be interpreted primarily with respect to the CT dimensions assessed by the CTt, particularly algorithmic thinking and structured problem-solving. In summary, CT has evolved from its origins in computer science education to become central to discussions of digital innovation and evolving educational settings. Current research emphasizes how new digital tools, such as cloud-based educational systems and interactive online platforms, empower students to engage in complex problem-solving activities and collaborate to develop knowledge [54]. In these technology-enhanced learning environments, CT operates as a mental framework that fosters abstraction, decomposition, and algorithmic thinking, rather than merely being a skill associated with programming [55,56]. These abilities are particularly important in today’s educational contexts, where students must tackle complex challenges and adapt to rapidly changing technological landscapes [57].

### Computational thinking and academic achievement

Recent research strongly underscores the crucial relation between students’ CT skills and their academic achievement across various fields [12,44,58,59]. By employing abstraction, algorithmic processing, and systemic thinking, computational thinking enables students to grasp concepts across multiple disciplines, including mathematics, science, and language studies [12]. CT helps in interpreting, analyzing, and solving various problems through abstraction, algorithms, and systemic thinking [43]. Evidence suggests that integrating CT into classroom practice enhances achievement and achievement-related constructs. For instance, according to Lodi and Martini, CT-based mathematics learning involves an interactive and cyclical process that integrates both mathematical and computational reasoning skills [47]. Moreover, a meta-analysis by Lei et al. highlights a significant relation between CT skills and achievement, indicating that improving these skills can enhance learning outcomes across various subjects [58]. On the other hand, Doleck et al. found no substantial connection, revealing that, aside from a negative correlation between cooperative and performance, other CT components such as algorithmic thinking, creativity, and problem-solving did not significantly predict academic results [36]. These differing findings emphasize the complex relationship between CT skills and academic achievement, suggesting the need for further exploration of the contextual factors involved.

### Computational thinking and psychological constructs

Deeper learning in CT occurs when individuals can transfer CT skills to different problem-solving contexts. This transfer process includes not only cognitive skills but also intrapersonal skills such as persistence and tolerance for ambiguity [60]. However, the research on the transfer of programming-related skills to far-removed domains such as higher-order thinking (problem solving, creative thinking) or psychological constructs (resilience, grit, growth mindset) remains rather limited. Gick and Holyoak demonstrated that one of the prominent components of CT, such as problem decomposition, is not easily transferable across different domains [61]. In line with this, the meta-analysis conducted by Scherer et al. reported strong near transfer for cognitive skills closely related to programming, while far transfer was generally found to be moderate to low [62]. In this context, resilience is implied as a potentially relevant intrapersonal attribute that may support the transfer of CT across domains [40]. Supporting this, the relationship between CT and individual traits such as psychological resilience and its potential mediating role in academic achievement has not yet been sufficiently explored. Within this framework, the present study seeks to advance the literature by proposing a novel model in which CT is examined as a mediating variable.

### The present study and research problems

Several theoretical mechanisms suggest that psychological resilience may be related to CT. CT has been conceptualized in the literature as a cognitive approach centered on abstraction and decomposition [13], later extended to include decomposition, abstraction, algorithm design, debugging, iteration, and generalization [63]. Together, these conceptualizations suggest that effective computational problem solving requires learners to continuously monitor, evaluate, and adapt their thinking and solution strategies. Self-regulated learning provides a theoretical explanation for this process by conceptualizing learning as a cyclical process in which learners actively engage in strategic planning, self-monitoring, and self-reflection to regulate their cognition and behavior while solving problems [64].

Executive function theory provides a complementary explanation. Diamond identified reasoning, planning, and problem solving as higher-order executive functions emerging from working memory, inhibitory control, and cognitive flexibility [65]. Similarly, Miyake et al. demonstrated that working memory updating, inhibitory control, and cognitive flexibility are distinct yet moderately correlated executive functions that make differential contributions to complex executive task performance [66]. Given the cognitive demands of CT described above, executive functions may provide an important cognitive foundation for successful engagement in CT. Accordingly, psychologically resilient learners may be better able to sustain these regulatory and executive processes when confronted with uncertainty, setbacks, and repeated failures, thereby facilitating the development of CT skills. In the present study, self-regulation and executive functions are therefore positioned not as competing focal mediators but as upstream theoretical processes that may help explain why CT can be related to psychological resilience. It should be emphasized that these upstream processes were not measured or tested in the present study; they serve solely as the theoretical background for the hypothesized resilience–CT association.

Building on this theoretical foundation, the present study selected CT as the focal mediating variable because because among the cognitive constructs theoretically linked to resilience, CT can be considered the more proximal pathway to mathematics and science achievement: whereas self-regulation and executive functions operate as domain-general regulatory resources, the algorithmic and structured problem-solving processes captured by the CTt are directly exercised in the kinds of tasks through which mathematics and science achievement is typically demonstrated. A further practical consideration supported this choice: CT could be assessed directly with a validated, performance-based instrument (the CTt) suitable for middle school students. Empirical findings also support the upstream regulatory account outlined above. Gao et al. demonstrated that self-regulation plays a central role in CT development by fully mediating the relationship between sequencing ability and CT [67]. Likewise, Zhou et al. reported that students with more sophisticated metacognitive profiles consistently demonstrated higher levels of CT and mathematical modeling competency, suggesting that higher-order regulatory capacities contribute to CT development [68]. More recently, Beyazhancer and Çepni demonstrated that CT partially mediated the relationship between mathematical modeling self-efficacy and flexible thinking, providing evidence that CT functions as a psychological bridge through which motivational beliefs are translated into adaptive cognitive outcomes [69]. Extending this line of research, Beyazhancer found that self-regulation partially mediated the relationship between psychological resilience and CT among pre-service teachers, offering preliminary evidence that resilience may contribute to CT through enhanced regulatory capacity [70]. These regulatory accounts suggest a concrete route from resilience to the specific CT processes measured in this study rather than a mere conceptual resemblance between the two constructs: persistence under difficulty may allow students to remain engaged with multi-step algorithmic tasks without disengaging prematurely; adaptive strategy revision parallels the testing and revising of conditional and loop structures; and sustained cognitive engagement after failure supports the repeated cycles of trial and correction required by sequence- and decision-based problems. On this account, resilience is expected not merely to resemble CT-related processes but to support their development and effective deployment. Notably, Wu et al. reported the reverse direction: CT instruction significantly enhanced students’ academic self-efficacy and academic resilience, providing further evidence that CT is closely associated with positive psychological and academic outcomes [71]. When considered alongside the theoretical accounts outlined above, this finding raises the possibility that resilience and CT may be reciprocally related. The present study examines one direction of this potential reciprocity—whether resilience is associated with achievement through CT—while acknowledging that the cross-sectional design cannot rule out reverse or bidirectional pathways. Collectively, these considerations suggest that the mixed findings on the direct resilience–achievement association may partly reflect the omission of intermediate cognitive pathways such as CT, among other possible factors such as differences in samples, measures, and educational contexts. Positioning CT as a focal mediator, therefore, provides a theoretically grounded and empirically testable specification of how psychological resilience may relate to mathematics and science achievement.

Accordingly, the present study is to examine the mediating role of CT in the relationship between psychological resilience and academic achievement in science and mathematics. The proposed model is presented in Fig 1.

**Fig 1:**
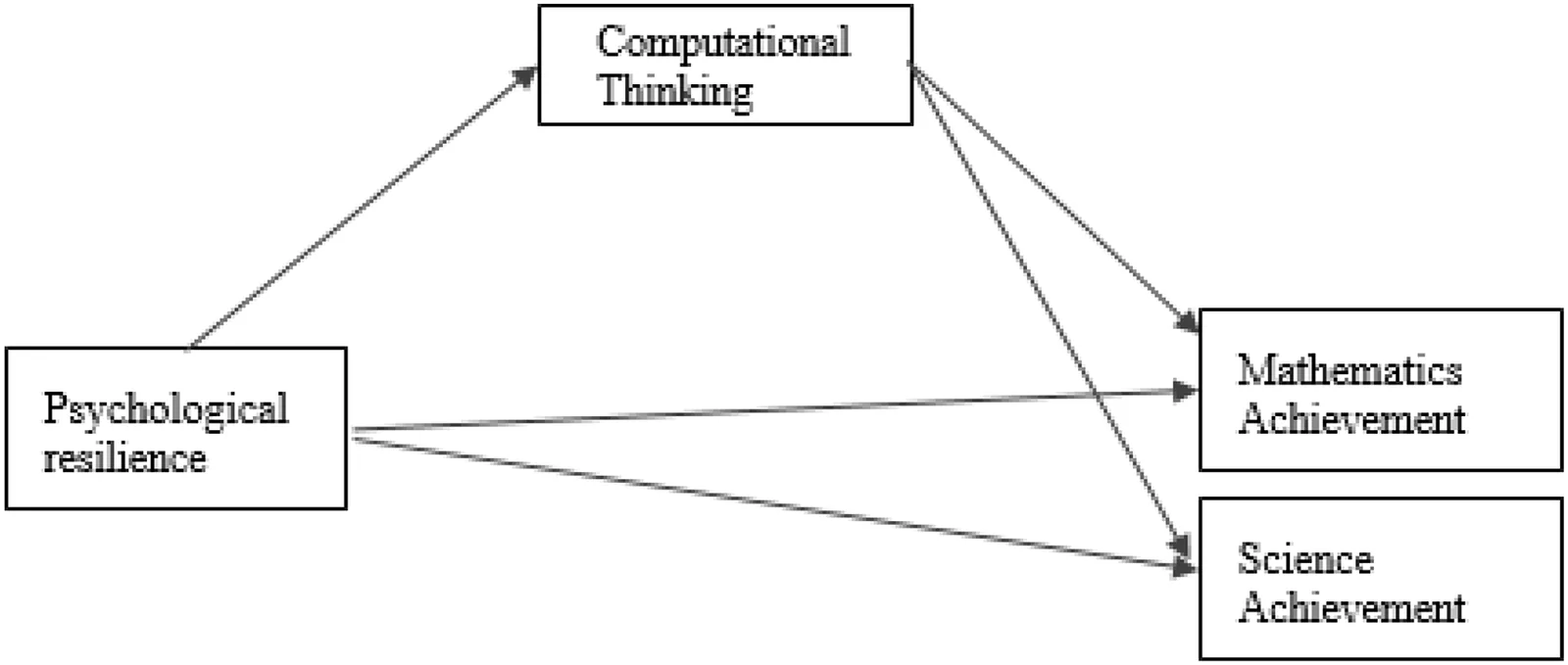
The path model is here.

This study poses the following research questions (RQs):

RQ1: Is there a significant relationship between psychological resilience, CT, and achievement in mathematics and science?

RQ2: Does CT mediate the relationship between psychological resilience and mathematics and science achievement?

## Methods

A correlational quantitative research design was used to examine the statistical mediation role of CT in the relationship between psychological resilience and academic achievement in mathematics and science [72]. Given the cross-sectional nature of the data, the results reflect statistical associations rather than causal inferences.

### Participants

The study sample of the study consists of middle school students at different grade levels attending public schools located in the Kocaeli and Bursa provinces of Türkiye. In the Turkish education system, Grades 5–8 correspond to lower secondary education (middle school level), which typically includes students aged approximately 10–14. Convenience sampling was employed based on accessibility, school permissions, and voluntary participation. This non-random approach is commonly used in school-based educational research, where institutional and ethical constraints make probabilistic sampling difficult. Although the method limits the generalizability of the findings, efforts were made to ensure diversity across grade levels, schools, and districts. A total of 401 students participated in the study on a voluntary basis. Of these students, 204 (50.9%) were female and 197 (49.1%) were male. In terms of grade levels, the distribution showed that 111 students (27.7%) were in the 5th grade, 116 students (28.9%) were in the 6th grade, 75 students (18.7%) were in the 7th grade, and 99 students (24.7%) were in the 8th grade. Data were collected between December 2024 and January 2025. Since the participants were middle school students under the age of 16, written informed consent was obtained from their parents or legal guardians prior to participation. Participation was voluntary, and participants were informed that they could withdraw from the study at any time without penalty.

### Instruments

#### Brief psychological resilience scale.

The Brief Psychological Resilience Scale (BRS), introduced by Smith et al. and subsequently validated in Turkish by Doğan, was employed to evaluate students’ levels of psychological resilience [73,74]. The instrument comprises a single-factor structure with six items, assessed using a 5-point Likert-type scale ranging from 1 (strongly disagree) to 5 (strongly agree). Items 2, 4, and 6 are reverse-coded. Higher scores indicate greater psychological resilience. The internal consistency coefficient (Cronbach’s alpha) was calculated as 0.83 for this study.

### Mathematics and science achievement

Students’ mathematics and science achievements were measured using their end-of-term course grades for the fall semester of the 2024–2025 academic year. These grades were based on students’ performance across multiple assessment components, including written examinations, homework assignments, projects, and other performance-based activities conducted during the semester. For each student, an overall achievement score was calculated as the arithmetic mean of these assessment results.

Grading was performed by subject teachers in line with the assessment and evaluation framework of the Turkish Ministry of National Education, using a scoring scale ranging from 0 to 100. The resulting scores were treated as continuous variables in the analysis. In total, eight mathematics teachers and six science teachers from the participating schools were involved in the grading process.

### Computational thinking test

The CTt, designed by Román-González and colleagues [51,53], evaluates students’ CT skills. Initially designed as a 40-item test, the assessment was later refined to 28 multiple-choice questions. The test measures seven core CT concepts, with four questions dedicated to each concept: simple functions, basic sequence structures, while loops, repeat loops, simple (if) decisions, repeat-until loops, and complex (if-else) decisions. The CTt was selected for the present study because it is a well-established, psychometrically validated instrument that is widely used to assess CT in educational settings. The test is closely aligned with the conceptualization of CT adopted in this study, particularly by assessing algorithmic thinking and problem-solving processes, which constitute core dimensions of CT. Therefore, the CTt was considered an appropriate instrument for measuring students’ CT skills in line with the objectives of the present study.

In the present study, the Turkish version of the CTt adapted by Sungur [75] was used. During the adaptation process, the test was translated from English to Turkish and back-translated to ensure linguistic equivalence. Content validity was evaluated through expert review involving specialists in science education, educational sciences, and language studies. The adapted version contains 25 items and maintains the original conceptual structure of the test. While these analyses provided initial evidence for the construct validity and reliability of the adapted instrument, further examination using item response theory approaches may offer additional insights into item functioning and measurement properties. Therefore, the present study employed Rasch modeling to obtain additional evidence of validity by examining item difficulty, person-item interactions, and potential differential item functioning across gender groups.

#### Rasch analysis.

The analysis was conducted using Winsteps 6.3.0, applying the Rasch model to assess item functioning across gender groups (male and female). Person measures ranged from −3.18 to 0.94 logits with a mean of −0.35 (SD = 1.12). Person separation was 1.71, and person reliability was 0.75, indicating moderate measurement precision. The item difficulties ranged from −1.55 to 1.80 logits with a mean of 0.61 (SD = 2.25). Item separation was 4.14, and item reliability was 0.94, suggesting that the sample was sufficient to establish item difficulty hierarchies. In addition, person and item fit statistics indicated satisfactory model fit. The mean Infit and Outfit MNSQ values for both persons and items were close to the expected value of 1.00. Table 1 summarizes the Rasch measurement statistics for persons and items.

**Table 1:** Summary statistics.

Summary of Measured Persons						Summary of Measured Items				
	Score	Measure	Realse	Infit MNSQ	Outfit MNSQ	Score	Measure	Realse	Infit MNSQ	Outfit MNSQ
*M*	10.0	-.35	.54	1.00	1.03	196.5	.61	.25	1.00	1.03
*SD*	4.3	1.12	.18	.21	.44	98.7	2.25	.47	.09	.20
Real RMSE			.57					.53		
True SD			.97					2.18		
Separation			1.71					4.14		
Reliability			.75					.94		

#### Gender-based DTF analysis.

Differential Item Functioning analysis was performed to identify whether individual items functioned differently across gender groups. The Mantel-Haenszel (MH) and Rasch-Welch tests were used to assess statistical significance. DIF statistics for each item, including DIF contrasts and effect sizes, were examined. Significant DIF was observed for Items 6 (p = .026), 7 (p = .018), and 25 (p = .016), suggesting that these items function differently for males and females. However, the DIF contrasts were below the threshold of 0.43 logits, indicating negligible DIF according to Linacre’s [76] criteria.

DTF was evaluated by aggregating DIF effects across all items to determine whether the test, as a whole, favored one gender over another. The chi-square test for overall DTF was not significant (χ² = 5.78, p = .8279), indicating that cumulative DIF effects did not bias the test. The item fit statistics, reliability indices, and negligible DIF findings provided additional support for the use of the total CT score in the subsequent mediation analyses. For visual inspection of the results, Fig 2 presents the person-item map (Wright Map), which shows the distribution of item difficulties and person abilities across gender groups.

**Fig 2:**
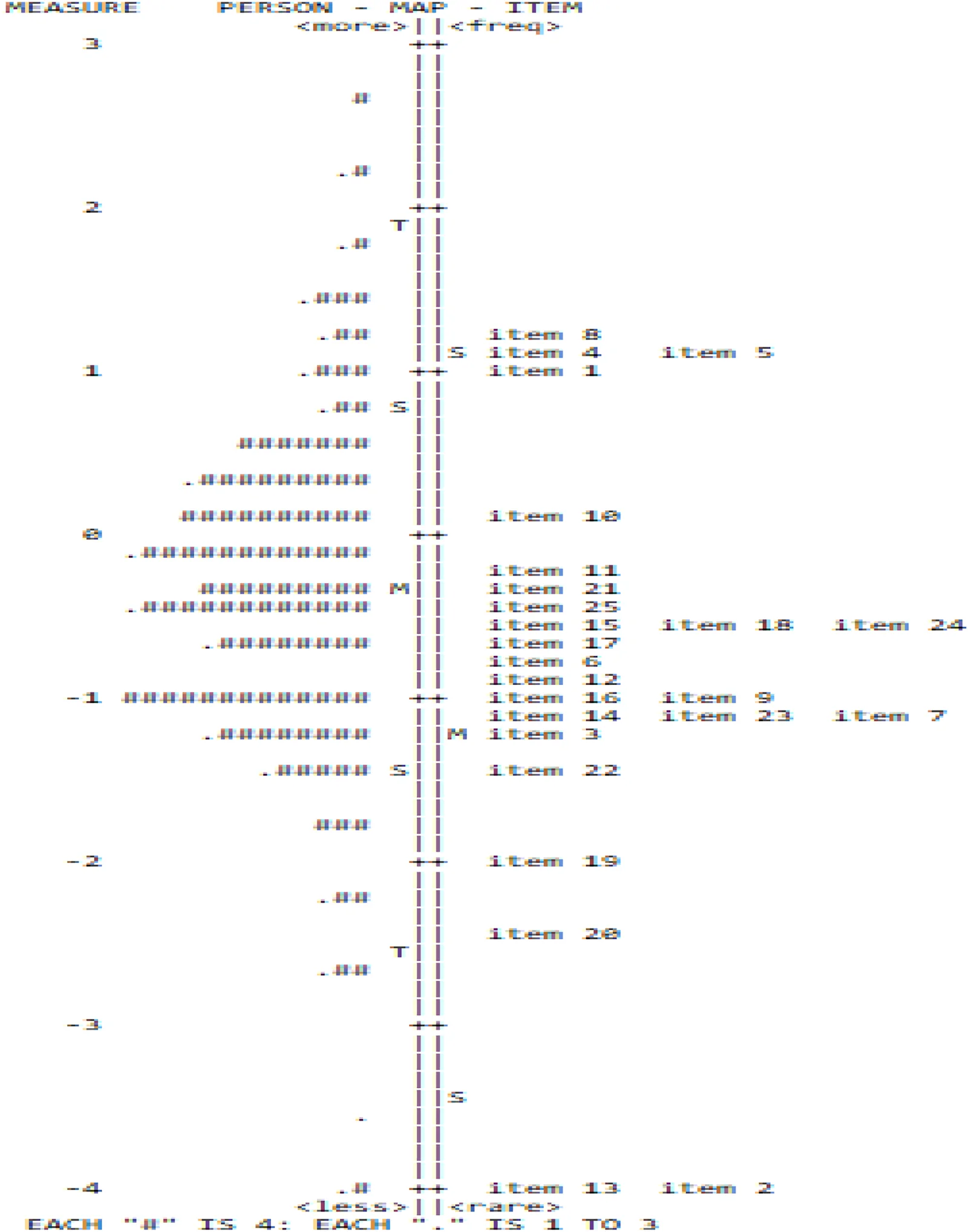
Distribution of Person Abilities and Item Difficulties is here.

### Research process and procedure

The research process was carried out in three main stages: ethical approval, participant briefing, and data collection.

### Ethical approval and permission process

All procedures performed in this study were approved by the Social and Human Sciences Research and Publication Ethics Committee of Bursa Uludağ University (Ethics Approval Number: E-92662996-044-181504) and conducted in accordance with the ethical standards of the institutional and national research committee and with the 1964 Helsinki Declaration and its later amendments or comparable ethical standards. Written informed consent was obtained from all participants after they were informed about the purpose, scope, and procedures of the study. In the case of participants under the age of 16, written informed consent was also obtained from their parents or legal guardians. The rights and confidentiality of all participants, especially minors, were strictly protected. All methods were carried out in full compliance with the relevant national and institutional guidelines and regulations.

### Participant briefing

Before data collection, the purpose and procedures of the study were explained to students in age-appropriate language.

### Data collection process

Data were collected online. The 25-item Computational Thinking Test (CTt) and the 6-item Psychological Resilience Scale were combined into a single Google Forms link. Students completed the form individually using computers in their schools’ information technology classrooms under teacher supervision. Each student was given approximately 40 minutes (one class period) to complete the form.

Several factors influenced the decision to use an online data collection method. First, because of the high number of items in the CTt and the inclusion of visual components (e.g., colors, shapes, and pixels), a printed version would have been both costly and technically challenging. The digital format preserved the original visual design of the test, thereby enhancing measurement validity. Additionally, online administration contributed to environmental sustainability by reducing paper consumption.

To enhance data quality, control questions were embedded within the form to ensure that students read the instructions carefully and responded consistently. Furthermore, response patterns were examined to identify potential careless responding, including straight-lining behavior (i.e., selecting the same response option repeatedly) and unusually rapid completion times. No systematic patterns indicating careless responses were detected.

To determine students’ academic achievement levels in mathematics and science, scores from written exams, homework, projects, and performance tasks completed during the 2024–2025 fall semester were used. These scores were officially obtained from school administrations, and an overall academic achievement score was calculated by averaging students’ semester performances. Scoring procedures followed the assessment and evaluation standards established by the Ministry of National Education (MoNE).

All data were collected and analyzed anonymously. Students’ identities were protected through the use of anonymous codes. The data were used solely for research purposes and were not shared with third parties. All procedures were conducted in accordance with the ethical standards of the 1964 Declaration of Helsinki and its later amendments.

### Data analysis

Data were analyzed using SPSS 28 for descriptive and correlational analyses and Mplus 8.1 for mediation. Descriptive and correlation analyses were conducted to examine variable distributions and relationships. Normality was assessed using skewness and kurtosis values, with thresholds of −2 to +2 indicating acceptable distribution [77]. A mediation analysis was conducted in Mplus, using maximum likelihood estimation with bias-corrected bootstrap confidence intervals based on 5000 resamples to examine the mediating role of CT in the relationship between psychological resilience and mathematics and science achievement. Gender was included in the model as a control variable, because prior studies have reported potential gender differences in CT [53,78,79]. Various fit indices were used to assess model fit, such as the Chi-square test (χ²), Root Mean Square Error of Approximation (RMSEA), Comparative Fit Index (CFI), Tucker-Lewis Index (TLI), and Standardized Root Mean Square Residual (SRMR). The model’s statistical fit was assessed using conventional cutoff values. The analysis examined the direct and indirect pathways linking psychological resilience to mathematics and science achievement through CT.

### Findings

#### Descriptive statistics.

The table presents the descriptive statistics and correlation coefficients among the variables analyzed in the study. Within the scope of the first hypothesis of the research, the skewness and kurtosis values of CT, science and mathematics achievement, and psychological resilience scale scores, as well as the correlations coefficients, were examined through correlation analysis; these results are presented in Table 2.

**Table 2:** Descriptive and correlations results of study variables.

	N	Mean	Std. Deviation	Skewness	Kurtosis	PR	CT	MA	SA
**PR**	401	3.15	0.63	.146	.919	1			
**CT**	401	10.82	3.88	.401	−.092	0.177**	1		
**MA**	401	75.77	20.81	−.755	−.312	0.130**	0.406**	1	
**SA**	401	80.49	16.53	−.938	.182	0.143**	0.372**	0.772**	1

PR = Psychological Resilience, CT = Computational Thinking, MA = Mathematics Achievement, SA = Science Achievement * p < 0.01, ** p < 0.001

Examining Table 2, we find that the calculated values fall within the specified range and meet the assumption of a normal distribution. The mean was score for psychological resilience is 3.15 (SD = 0.63). For CT, the scores ranged from 2 to 21; the mean is 10.82 (SD = 3.88) out of a possible 25 points. This corresponds to approximately 43% of the maximum attainable score, which suggests that students demonstrated a moderate level of CT performance on the test. The mean score for science achievement is 80.49 (SD = 16.53). The average score for mathematics achievement is 75.77 (SD = 20.81).

RQ1: Is there a significant relationship between psychological resilience, CT, and achievement in mathematics and science?

To answer this research question, Pearson correlation analyses were conducted on the main variables: psychological resilience, CT, math achievement, and science achievement. The results, presented in Table 2, indicate statistically significant positive relationships among all variables. The magnitude of the correlations was interpreted using Cohen’s guidelines for effect size [80**]**. According to these benchmarks, correlations around.10 indicate small effects, around.30 indicate moderate effects, and around.50 or higher indicate large effects. Specifically, psychological resilience was positively correlated with CT (r = .177, p < .01), mathematics achievement (r = .130, p < .01), and science achievement (r = .143, p < .01). Moreover, CT was moderately and positively associated with both mathematics (r = .406, p < .01) and science achievement (r = .372, p < .01). These findings suggest that students with higher levels of psychological resilience tend to exhibit stronger CT skills, and both resilience and CT are positively associated with academic success in mathematics and science. Based on these significant associations, further analyses were conducted to explore the potential mediating role of CT in the relationship between psychological resilience and academic achievement.

### Measurement model verification

In the present study, a path analysis approach has been employed to systematically examine the relationships among psychological resilience, CT, and academic achievement in mathematics and science. In line with the proposed theoretical framework, the analysis first examined the direct effects of psychological resilience on mathematics and science achievement. Subsequently, the potential mediating role of CT in this relationship was examined to determine whether CT statistically mediated the association between psychological and academic performance. The role of CT as a mediator has been considered without including any moderator variables in the model. Potential multicollinearity among the predictors was assessed using variance inflation factors (VIFs) and tolerance values. All VIF values were below 5, and tolerance values exceeded.20, indicating no serious multicollinearity.

RQ2: Does CT mediate the relationship between psychological resilience and mathematics/science achievement?

To investigate the relationship between psychological resilience and the mathematics and science achievement of middle school students, taking into account the mediating effect of CT, parallel mediation analyses were conducted for these outcomes. In the mediation model, mathematics achievement and science achievement were treated as two separate outcome variables. This allowed the relationships among psychological resilience, CT, and academic performance to be examined independently for each subject domain. To control for the effect of gender, gender was also included in the model. The model was analyzed using maximum likelihood estimation. The structural relationships among the variables are presented in Fig 3.

**Fig 3:**
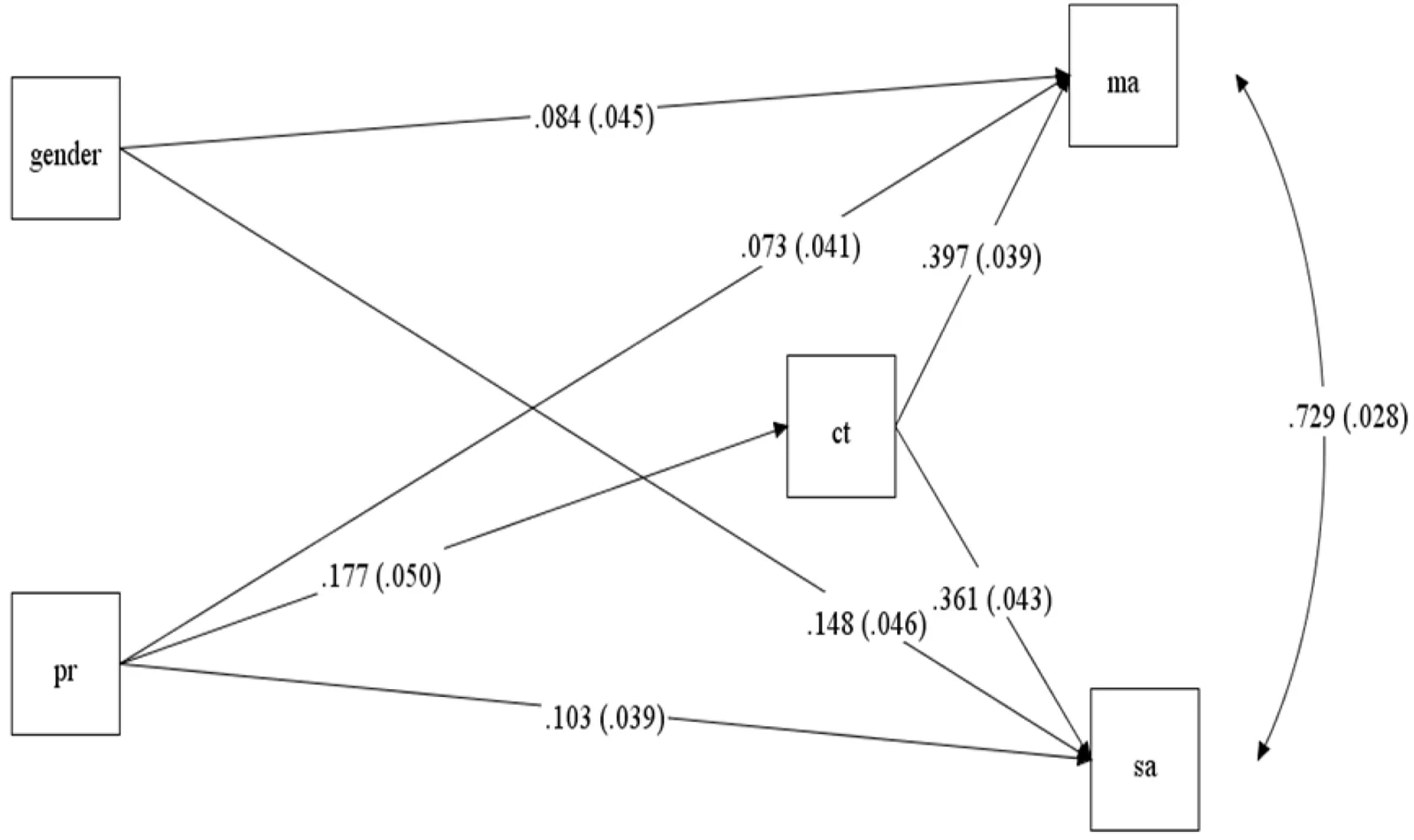
Mediation model of the relationship between psychological resilience (pr), computational thinking (ct), and academic achievement in mathematics (ma) and science (sa) is here.

Fit indices such as RMSEA, CFI, TLI, and SRMR, and the Chi-square (χ²) test were used to evaluate the fit of the model to the data. The results of the chi-square test (χ² = 0.268, df = 1, p = 0.605) provide a good fit. The RMSEA was 0.000 (90% CI: 0.000–0.106), which indicated that the model exhibited a near-perfect fit. The CFI (1.000) and TLI (1.014), which are close to or exceed 1.0, further support the good fit of the model. Moreover, the SRMR value of 0.008 indicates that the standardized residuals are minimal and the model’s internal consistency. Overall, these indices indicate that the specified path model showed very good fit to the observed data [81]..

According to the results of the analysis, CT was positively and significantly associated with both mathematics and science achievement. The association between CT and mathematics achievement was quite strong (𝛽 = 2.130, p < 0.001), and its effect on science achievement was also significant (𝛽 = 1.539, p < 0.001). Psychological resilience was positively and significantly associated with CT (𝛽 = 1.091, p < 0.001). However, the association between psychological resilience and mathematics achievement was not statistically significant (𝛽 = 2.401, p = 0.076), while its effect on science achievement was statistically significant (𝛽 = 2.695, p = 0.010). The gender variable was significantly associated with science achievement (𝛽 = 4.898, p = 0.002), while its association with mathematics achievement was not significant (𝛽 = 3.502, p = 0.067).

Regarding the indirect associations between psychological resilience and mathematics and science achievement through CT, the analysis revealed significant indirect pathways. Specifically, higher levels of psychological resilience were associated with increased CT skills, which, in turn, were positively associated with both science and mathematics achievement. This mediation effect was statistically significant for both science (β = 0.064, p = .001) and mathematics (β = 0.070, p = .001). Since the direct association between psychological resilience on mathematics achievement was not statistically significant, the findings were statistically consistent with CT mediating this relationship. In contrast, psychological resilience showed both a significant direct association and a significant indirect association with science achievement, which was statistically consistent with partial mediation. The unstandardized and standardized coefficients for the mediation model are presented in Table 3.

**Table 3:** The unstandardized and standardized coefficients for the mediation model.

Unstandardized coefficients					Standardized coefficients				
**Effect Estimate**		**95%CI**	**s.e**	**p**	**Estimate**	**95%CI**	**s.e**	**p**	**R2**
On mathematics achievement									0.18
gender	3.50	−0.25–7.25	1.915	0.067	.084	−0.005-0.18	0.045	0.064	
CT	2.13	1.72–2.54	0.207	<.001	.397	0.32-0.47	0.039	<.0001	
psychological resilience	2.40	−0.25-5.05	1.353	0.076	.073	−0.007-0.153	0.041	0.073	
Indirect effect	2.35	0.97-3.68	0.692	0.001	.070	0.036-0.11	0.021	0.001	
On science achievement									0.17
gender	4.90	1.87-7.93	1.545	<.001	.148	0.059-0.237	0.046	0.001	
CT	1.54	1.18- 1.90	0.184	<.001	.361	0.28-0.45	0.043	<.0001	
psychological resilience	2.70	0.63-4.76	1.052	.01	.103	0.03-0.18	0.039	0.009	
Indirect effect	1.68	0.67-2.69	0.517	.001	.064	0.026-0.102	0.019	.001	

p < 0.01, p < 0.001

## Discussion

The present study investigated the relationship between psychological resilience and academic achievement in science and mathematics as well as the mediating role of CT in that relationship. First of all, according to the findings, CT was positively related to not only mathematics but also science achievement. This was not a surprising finding since the relevant literature also suggests a connection between CT skills and academic success across disciplines [12,44,58]. In other words, students who have the ability to abstraction, algorithmic thinking, or system thinking may solve complex problems in mathematics and science more effectively [12,43]. Notably, Lei et al.‘s meta-analysis supports this by demonstrating a significant positive correlation between CT and academic performance, suggesting that higher levels of these skills are associated with better learning outcomes [58]. Furthermore, Lodi and Martini found that CT-based instructional approaches, such as those integrating mathematical and computational reasoning deepen conceptual understanding and improve achievement [47]. However, it is important to acknowledge that the relationship between CT and academic success is not universally consistent, as some studies, such as Doleck et al., have found limited predictive power of certain CT components [36]. These discrepancies highlight the need for further research into contextual factors influencing this relationship. For instance, Scherer et al. emphasize that CT skills developed through programming often show weak far-transfer effects to abstract academic domains [62]. They also suggested that while CT is powerful when integrated into domain-specific learning (e.g., mathematics problem solving), its influence may be less pronounced when disconnected from instructional context. Thus, the integration of CT into learning environments must be intentional and pedagogically grounded to produce measurable outcomes in academic performance.

Regarding psychological resilience, the results indicate that it was positively associated with mathematics and science achievement, although the effect size was small. In other words, the findings suggest that students who have the ability to adapt to and recover from challenges tend to achieve greater success in mathematics and science than their peers. This is an expected finding, as the previous literature also suggests that students with psychological resilience may have higher academic success [3]. On the other hand, according to the results, psychological resilience is positively related to mathematics achievement and science achievement, even though both correlations fall within the small-effect range according to Cohen’s benchmarks [80]. The modest strength of the relationship strengthens the idea that psychological resilience alone is not a dominant predictor of achievement. Instead, it is likely to interact with other factors.

This view is supported by several studies that question the predictive strength of resilience across all contexts. For instance, Hudson and Reynoso found that psychological resilience did not significantly predict academic achievement in some cases, suggesting that its effect may be domain-dependent or mediated by other competencies [27,28]. In this respect, CT may represent one possible pathway linking psychological resilience to achievement, particularly in problem-centered tasks. The current study also examined the mediating role of CT in the relationship between psychological resilience and mathematics and science achievement. The findings were statistically consistent with a pattern in which the direct association between psychological resilience and mathematics achievement became non-significant after accounting for CT, whereas a significant direct association remained for science achievement. These results suggest that the association between psychological resilience and academic achievement may be statistically explained, in part, through CT. In particular, for mathematics achievement, the non-significant direct association and the significant indirect association were statistically consistent with CT playing a mediating role. This finding is consistent with previous research suggesting that the association between resilience and academic achievement may depend on other cognitive characteristics, such as CT [33].

However, the small correlations involving psychological resilience and the modest proportion of explained variance indicate that resilience is not a dominant predictor of achievement, and that CT represents only one possible statistical pathway. Moreover, unmeasured factors, such as prior achievement, socioeconomic status, motivation, and school or teacher effects, may have influenced the estimated mediation paths. Because the nesting of students within classrooms and schools was not modeled, these findings should be interpreted cautiously as conditional associations within the specified model.

Nevertheless, the present findings may also be consistent with alternative directional explanations. For example, students with higher levels of mathematics and science achievement may develop stronger CT skills through sustained engagement with discipline-specific problem-solving experiences [82]. Similarly, reciprocal relationships between cognitive skills and academic achievement may emerge over time, with each reinforcing the other through developmental processes [83,84]. Therefore, the model proposed in the present study should be interpreted as one plausible theoretical explanation rather than the only possible account of the observed associations.

In addition, an important nuance emerged in how CT mediated the relationship between psychological resilience and achievement. Although the findings were statistically consistent with no significant direct association with mathematics achievement after accounting for CT, a significant direct association remained for science achievement. This finding suggests that different mechanisms may underlie achievement across domains. The finding that the results were statistically consistent with full mediation for mathematics achievement may reflect the close alignment between CT and the structured problem-solving processes that characterize mathematical tasks [71]. In contrast, findings statistically consistent with partial mediation in science suggest that the association between psychological resilience and science achievement may also involve additional pathways beyond CT. One possible explanation for this domain-differential pattern can be the pedagogical and epistemic characteristics of mathematics and science learning contexts. Mathematics learning typically emphasizes abstract reasoning, symbolic manipulation, and structured, algorithmic problem-solving processes that closely align with core components of CT, such as decomposition, abstraction, pattern recognition, and algorithmic thinking [85]. In line with this, recent studies in the international literature have highlighted the role of CT in enhancing students’ problem-solving skills and academic achievement, emphasizing its function as a cognitive framework that supports structured reasoning and knowledge transfer across domains [55,86]. Within such contexts, higher psychological resilience may be associated with greater engagement in CT-related problem-solving processes during complex mathematical tasks. In contrast, science learning often involves inquiry-based practices, experimentation, and engagement with empirical phenomena, where students may encounter uncertainty, unexpected results, or experimental failure [87,88]. In these situations, psychological resilience may also be associated with persistence and engagement beyond purely computational or algorithmic reasoning processes.

This distinction is also reflected in the Turkish educational context. The national mathematics curriculum emphasizes a transition toward abstract reasoning and algorithmic problem-solving [89], which may channel students’ resilience primarily through CT-related cognitive strategies. In contrast, the Turkish science curriculum places a strong emphasis on inquiry-based learning [90]. Therefore, resilience may be associated with science achievement through both CT-related pathways and students’ ability to cope with the inherent challenges of inquiry-based learning environments. Furthermore, science is a multifaceted domain [87], where resilience may support persistence in experimental and collaborative contexts independent of CT. This interpretation is consistent with recent bibliometric evidence suggesting that CT has evolved from a programming-oriented construct to a broader cognitive framework in mathematics education [91]. Accordingly, the findings, which were statistically consistent with a full mediation pattern for mathematics achievement, contribute to the growing recognition of CT as an important cognitive skill set in mathematical learning.

The originality of the present study lies in its integrative perspective, linking a psychological trait (resilience) with a cognitive skill set (CT) within the context of domain-specific academic performance. It extends the literature by suggesting that the association between psychological resilience and achievement may be statistically explained, in part, through CT, particularly in mathematics. These findings highlight CT as a potential explanatory link between affective strengths and cognitive performance. These findings suggest that learning environments aimed at supporting mathematics and science achievement may consider the potential relevance of psychological resilience and CT. However, whether educational practices targeting these constructs can improve academic achievement remains an empirical question that should be examined in future intervention-based research. Given the characteristics of the CTt employed in the present study, the observed mediating effect is more closely related to core CT processes, particularly algorithmic thinking, abstraction, and structured problem-solving. However, because the present study did not examine CT sub-dimensions separately, it is not possible to determine which specific CT sub-dimension contributes most strongly to this relationship.

### Limitations and future directions

This study has several limitations. Firstly, it is a correlational and cross-sectional study. Therefore, it does not aim to make causal inferences. Accordingly, any reference to an “effect” in the statistical analysis refers to a statistical effect estimate and should not be interpreted as evidence of a causal effect. Future longitudinal and experimental studies are needed to examine the temporal ordering and potential causal relationships among psychological resilience, CT, and academic achievement. Second, the study was conducted with middle school students from public schools in two specific provinces (Kocaeli and Bursa, Turkiye). This approach is common in school-based educational research, but it may limit how broadly the findings can be generalized. Students’ socioeconomic and educational traits may differ from those in other areas. The study did not directly measure socioeconomic status, a factor frequently associated with resilience and academic success. Therefore, unmeasured differences in socioeconomic status could have influenced the observed relationships. Future research should use more diverse and representative samples from different geographic areas and include socioeconomic indicators. This may contribute to a deeper understanding of how inequalities in the environment affect resilience and academic outcomes.

In addition, mathematics and science achievement were based on official school grades assigned by multiple teachers across different schools. Although all teachers followed the assessment framework established by the Turkish Ministry of National Education, grading practices may still have varied across teachers and schools. Such variability may have introduced additional measurement error into the achievement scores and should be considered when interpreting the findings.

Although the total CTt score provided a valid and reliable measure for the present mediation analysis, and the seven computational concepts were treated as indicators of a single overall construct, these concepts could in principle be examined as separate facets of CT The present study did not examine the sub-dimensions in separately; it therefore remains possible that some facets of CT (e.g., algorithmic thinking versus abstraction) contribute more strongly to the mediating relationship than others. Future research should consider analyzing CT sub-dimensions individually, guided by a priori hypotheses regarding which specific CT processes are expected to mediate the association between psychological resilience and academic achievement in mathematics and science.

Finally, although the model included gender as a control variable, it did not include several other variables that may simultaneously influence psychological resilience, CT, and academic achievement, such as socioeconomic status, prior achievement, school context, teacher effects, and access to technology. Their omission may have biased the estimated direct and indirect paths in the mediation model. In addition, the mediation model did not account for other potential mediators (e.g., self-efficacy, motivation) or moderators (e.g., socioeconomic status, teacher support) that might influence the relationships examined. Moreover, the partial mediation for science achievement suggests that unmeasured variables may play a role and should be further investigated. Future research should explore the dynamic interactions among CT, resilience, and other contextual variables such as socioeconomic background, self-efficacy, and metacognitive awareness. Longitudinal or intervention-based studies can help clarify the causal mechanisms and provide stronger evidence for targeted educational strategies across STEM domains.

## Conclusion

This study examined whether CT mediates the association between psychological resilience and academic performance in mathematics and science among Turkish middle school students. The findings suggest that psychological resilience may be associated with academic achievement and that this association is statistically consistent with a mediating role of CT. A significant finding was that mediation patterns differed across the two academic domains. Specifically, the findings were statistically consistent with a full mediation pattern involving CT. This pattern is consistent with the possibility that CT-related systematic and sequential problem-solving processes may be relevant to the observed association between psychological resilience and mathematics achievement. For science achievement, the findings were statistically consistent with a partial mediation pattern involving CT. This pattern suggests that the association between psychological resilience and science achievement may involve CT-related cognitive processes as well as other factors. One potential explanation is that science learning frequently entails inquiry-based activities, experimentation, and collaborative problem-solving, which could necessitate skills beyond computational reasoning. From a pedagogical perspective, the findings highlight the potential relevance of considering both cognitive skills and psychological resilience in STEM education. However, whether educational practices targeting CT and psychological resilience can improve learning
